# Menstrual cycle influence on skeletal muscle mitochondrial respiration in humans

**DOI:** 10.14814/phy2.70458

**Published:** 2025-07-12

**Authors:** W. Bradley Nelson, Brandon S. Pfeifer, Ashley R. Lovell, Erik D. Marchant, Briell V. Mitchell, Erin L. Atkinson, Trevor L. Murphy, Sydney L. Poulsen, Jay R. K. Baird, Chad R. Hancock, Robert D. Hyldahl

**Affiliations:** ^1^ Exercise Sciences Brigham Young University Provo Utah USA; ^2^ Nutrition, Dietetics and Food Science Brigham Young University Provo Utah USA

**Keywords:** menstrual cycle phase, mitochondrial respiration, skeletal muscle

## Abstract

The menstrual cycle influences function in various tissues in the body. We sought to determine if menstrual cycle phase could influence mitochondrial function in skeletal muscle in females. Twenty‐nine females with regular menstrual cycles were randomized to have a vastus lateralis muscle biopsy during either the early follicular or luteal phase. High‐resolution respirometry was used to determine mitochondrial respiration on permeabilized muscle fibers. Glutamate/malate LEAK respiration was significantly higher during the luteal phase compared to the early follicular phase. Glutamate/malate/succinate LEAK respiration was the same during both menstrual cycle phases, as was maximal coupled and uncoupled respiration. There were no differences in fatty acid‐supported respiration. The fatty acid‐supported coupling efficiency ratios of 1‐OcM (octanoylcarnitine/malate) LEAK over maximal coupled respiration and 1‐OcM LEAK over maximal uncoupled respiration were both significantly higher in mitochondria from the early follicular phase than in the luteal phase. Mitochondrial H_2_O_2_ emission (glutamate/malate/succinate supported) was significantly increased in muscle from the early follicular phase. We detected no differences in mitochondrial content using citrate synthase activity between phases of the menstrual cycle. Collectively, our observations demonstrate a limited influence of the menstrual cycle on certain measures of submaximal respiration, coupling efficiencies, and H_2_O_2_ emission.

## Graphical abstract

The influence of the menstrual cycle was examined and found to influence elements of mitochondrial respiration and hydrogen peroxide release but not maximal respiration or citrate synthase activity.

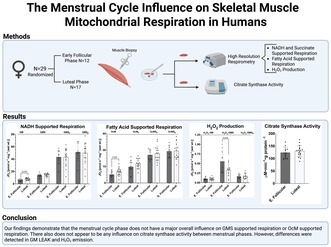

## 1 INTRODUCTION

Mitochondria play essential cellular roles in ATP synthesis, reactive oxygen species generation, apoptosis, calcium management, and other vital functions. Meanwhile, mitochondria located in skeletal muscle play fundamental roles in regulating several key factors in overall health. Improvements in mitochondrial respiratory capacity and content in skeletal muscle coincide with enhanced insulin sensitivity, preservation of muscle mass, and endurance exercise performance (Benton et al., 2008; Dodds et al., 2018; Fitts et al., 1975). Mitochondrial dysfunction, as characterized by changes in morphology, content, electron transport function, and reactive oxygen species generation, is associated with numerous pathologies including muscle atrophy, insulin resistance, and diabetes (Brown et al., 2017; Buso et al., 2019; Kavazis et al., 2009; Morton et al., 2019; Pinti et al., 2019; Powers et al., 2011; Remchak et al., 2021; Sangwung et al., 2020). As such, understanding the influences of normal physiological phenomena on mitochondrial function is necessary to better understand mitochondria and their overall role in health and disease.

The menstrual cycle, an umbrella term for the collective ovarian and uterine cycles, is a fundamental element of female physiology that characterizes female reproductive function. The ovarian cycle regulates oocyte development and release and is comprised of three phases: the follicular, ovulatory, and the luteal. Occurring roughly every 29 days (Bull et al., 2019), this rhythmic cycle is regulated by phasic changes in circulating pituitary hormones (follicle stimulating hormone and luteinizing hormone) and ovarian hormones (estradiol and progesterone) (Reed & Carr, 2000). These phasic variations in circulating hormones are responsible for various physiological changes throughout the cycle. For example, the menstrual cycle induces phasic changes in immune function, temperature regulation, skin function, and vascular function, as reviewed here (Farage et al., 2009). Metabolically, the menstrual phases are also reported to influence both protein intake (Gorczyca et al., 2016) and insulin sensitivity (Yeung et al., 2010). In females with type I diabetes, the risk of hyperglycaemia was also associated with the phase of the menstrual cycle (Brown et al., 2015). These systemic influences are attributed to the circulating hormones and the presence of ovarian hormone receptors found on target tissues.

Work in cell culture, rodents, and humans has demonstrated that ovarian hormones can influence mitochondrial function in skeletal muscle (Gras et al., 2007; Kane et al., 2011; Ribas et al., 2016; Torres, Kew, et al., 2018; Torres, Ryan, et al., 2018). Given this, it is plausible that mitochondrial function may be influenced by the natural cyclical fluctuations of these hormones throughout the menstrual cycle. However, to date, there are no existing reports of mitochondrial function across phases of the menstrual cycle. As such, we set out to describe the influence of normal menstrual cycle phases on skeletal muscle mitochondrial function. We examined two time points in the menstrual cycle characterized by considerable differences in circulating ovarian hormones, the early follicular phase, notable for its relatively low levels of circulating ovarian hormones, and the luteal phase, characterized by elevated ovarian hormones. We hypothesized that the luteal phase of the menstrual cycle would increase mitochondrial respiration and decrease reactive oxygen species emission.

## 2 MATERIALS AND METHODS

### 2.1 Ethics approval

The study was conducted in compliance with the current Declaration of Helsinki of 1975 (except for registration in a database) and approved by the Institutional Review Board at Brigham Young University (IRB# 2021‐132). All participants were informed of the experimental procedures and risks and provided written informed consent.

### 2.2 Participant characteristics

Twenty‐nine healthy, young (18–25 years of age) female volunteers participated in the study. This study is a companion study and includes data generated from participants from another investigation that reported the influence of menstrual cycle phase on eccentric muscle damage (Pfeifer et al., 2024); the data contained in this report are unique to this study, and there is no duplication. Participants self‐reported that they engaged in less than 2 h per week of physical activity with no regular resistance training during the 12 months prior. Furthermore, participants were screened and excluded for irregular menstrual cycles, pregnancy, use of hormonal contraceptives, smoking, prescription medications within the preceding 6 months, and current use of pain medications (i.e., nonsteroidal anti‐inflammatory drugs, opioids, and acetaminophen). Participant characteristics are further detailed in Table 1.

**TABLE 1:** Participant characteristics.

Group	Sample size	Test day	Menstrual cycle length (days)	Age (years)	Height (cm)	Body mass (kg)	BMI
E. Follicular Phase	12	3.5 ± 1.6	29.0 ± 2.2	20 ± 2	167 ± 11	63 ± 11	22 ± 2
Luteal Phase	17	25.2 ± 3.4	29.3 ± 2.2	22 ± 2	165 ± 8	59 ± 8	21 ± 1

*Note*: Menstrual cycle length, mean length of the preceding six menstrual cycles. Test day, day of the menstrual cycle participants underwent muscle biopsy and mitochondrial respiration measurements were made. All values presented as mean ± standard deviation. Differences in means for menstrual cycle length, height, and BMI were tested using unpaired *t*‐tests. Differences in means for age and body mass were tested with Mann–Whitney *U* tests. Sample sizes: Early follicular *n* = 12, luteal *n* = 17.

Abbreviations: BMI, body mass index; E. Follicular, early follicular.

### 2.3 Study description

Study participants were randomly assigned using a computer randomizing procedure (random.org/lists) that operated under a simple randomization protocol and was performed for each participant after recruitment and prior to data collection. Participants were randomly assigned to be tested during either the early follicular phase (*n* = 12) or luteal phase (*n* = 17) of the menstrual cycle. Originally, there were 14 participants in the follicular group and 15 in the luteal phase group, but after data collection was finished and reviewed, it was discovered that two participants of the follicular group were biopsied during the luteal phase of their menstrual cycles. As such, these two participants were moved to the luteal phase group. Prior to participating in the study, participants discussed with study personnel their previous six menstrual cycles to ensure that participants experienced consistent menstrual cycles (fewer than 6 days variance) to facilitate participant visits to the lab for data collection. Participants were provided with commercially available luteinizing hormone (LH) ovulation test kits (Premom, Easy Healthcare Corporation, Burr Ridge, IL) to track the mid‐menstrual cycle ovulatory event. Ovulation was assumed to occur 2 days following the LH surge detected by the kits. Once ovulation was determined, participants were then scheduled for their biopsy lab visit during either the early follicular phase (within first 7 days after beginning menses) or luteal phase (after ovulation but before menses) of their menstrual cycle, in accordance with their randomly assigned group.

### 2.4 Lab visit

Participants arrived at the lab on their scheduled day that coincided with their randomized menstrual cycle phase. A random leg was selected for a percutaneous muscle biopsy. A muscle biopsy was taken from the vastus lateralis muscle using the Bergström technique, as described here (Shanely et al., 2014). Chlorhexidine was used to sterilize the skin, and the area was anesthetized with 1% lidocaine with epinephrine. A 1 cm incision was made in the skin and underlying muscle. A Bergström biopsy needle was inserted about 3 cm into the muscle. A roughly 75–150 mg sample of muscle was removed using manual suction. About 20 mg of the muscle sample was placed in an ice‐cold BIOPS buffer (60 mM K‐MES, 35 mM KCl, 7.23 mM K_2_EGTA, 2.77 mM CaK_2_EGTA, 20 mM imidazole, 0.5 mM DTT, 20 mM taurine, 5.7 ATP, 15 mM PCr, and 6.56 mM MgCl_2_) to measure muscle mitochondrial respiratory capacity.

### 2.5 Mitochondrial respiration

Small bundles of skeletal muscle fibers (2–5 mg) were delicately teased apart with fine‐tipped forceps to partially separate fibers, blotted dry, and then weighed (2–5 mg). Fiber bundles were rotated at 4°C for 30 min in BIOPS buffer supplemented with 50 μg·mL^−1^ saponin to selectively permeabilize the sarcolemma. After permeabilization, bundles were rinsed for 15 min in ice‐cold MiR05 buffer (110 mM sucrose, 60 mM potassium lactobionate, 2 mM MgCl_2_, 20 mM taurine, 10 mM KH_2_PO_4_, 0.5 mM EGTA, 20 mM HEPES, and 1 g·L^−1^ bovine serum albumin). After rinsing, bundles were set inside an Oroboros Oxygraph‐2 k (Oroboros Instruments GmBH, Innsbruck, Austria) with MiR05 buffer at 37°C while the stir bars were set to 750 revolutions·minute^−1^. Supplemental O_2_ was used to maintain O_2_ concentrations between 200 and 500 μM during all measurements. All measures were made in duplicate, due to variability in the preparation phases (e.g., weighing and teasing bundles), and then averaged. Mitochondrial substrates were added using the following two titration protocols to measure mitochondrial respiration:

#### 2.5.1 Protocol 1

Glutamate (10 mM final concentration, Millipore Sigma G1626) and malate (2 mM, Millipore Sigma M1000) were first added to stimulate GM LEAK respiration. Succinate (10 mM, Millipore Sigma S2378) was subsequently added to maximally stimulate GMS LEAK respiration as described by Pesta and Gnaiger (2012). ADP was added in three steps (12.5 μM, 25 μM, and 5 mM; Millipore Sigma A528) to evaluate the effect of a small increase in ADP on respiration and mitochondrial reactive oxygen species production. The highest concentration of ADP (5 mM) is considered to be supraphysiological and represents maximum or near maximum ADP stimulating conditions (GMS_P_). Mitochondrial membrane integrity was verified by the addition of cytochrome‐*c* (10 μM, Millipore Sigma C7752). Increases in O_2_ consumption greater than 15% upon the addition of cytochrome‐*c* were seen as a positive indicator that the mitochondrial membrane integrity had been lost and the respective sample was excluded from further analysis. Maximal uncoupled respiration (GMS_E_) was evaluated by titrating FCCP (0.5 μM increments, Millipore Sigma C2920) which allows protons to cross the inner membrane independent of ATP synthase. Complex III was then inhibited with antimycin A (2.5 μM, Millipore Sigma A8674) to determine residual O_2_ consumption from complex III independent pathways. Residual O_2_ consumption was subtracted from previous measures.

#### 2.5.2 Protocol 2

OcM LEAK respiration was stimulated by the addition of octanoylcarnitine (0.5 mM, ApexBio B6371) and malate (2 mM). Maximal fatty acid oxidation (OcM_P_) was stimulated by the addition of ADP (2.5 mM) which was followed by the addition of 10 mM succinate (OcMS_P_). Mitochondrial membrane integrity was verified by the addition of 10 μM cytochrome‐*c*. Maximal uncoupled fatty acid oxidation (OcMS_E_) was induced by the addition of FCCP; antimycin A was then added (2.5 μM, Millipore Sigma A8674) to establish background O_2_ consumption from complex III independent pathways. O_2_ consumption (*J*O_2_) is expressed in pmol O_2_·s^−1^·mg wet tissue mass^−1^.

After acquisition, all respiration traces were evaluated for consistency and expected responses to titrations. If deviations from established trends were observed, the data were further reviewed. If protocol abnormalities were detected (e.g., insufficient tissue mass and technical issues with titrations) the data were excluded from final analysis.

### 2.6 Mitochondrial H_2_O_2_ production

The fluorimetry module of the Oroboros Oxygraph‐2 k was used to determine the rate of mitochondrial H_2_O_2_ production (*J*H_2_O_2_) (Makrecka‐Kuka et al., 2015). Prior to the addition of permeabilized muscle tissue, a standard curve was created using Amplex Red (10 μM, ThermoFisher Scientific A12222) and horseradish peroxidase (1 U·mL^−1^, Millipore Sigma P8250). Titrations of 0.1 μM H_2_O_2_ were added in steps. H_2_O_2_ reacts with Amplex Red and is converted to resorufin, catalyzed by horseradish peroxidase. The resorufin signal is detected due to its fluorescence. H_2_O_2_ was then measured in muscle samples in tandem with the O_2_ consumption analysis. *J*H_2_O_2_ is expressed in pmol H_2_O_2_·s^−1^·mg wet tissue^−1^.

### 2.7 Citrate synthase activity

To determine citrate synthase activity, whole muscle homogenate was added to 1 mL cuvettes with 25 μL of 10% Triton X‐100, 25 μL of 12.2 mM Acetyl‐CoA, 100 μL of 1.01 mM DTNB, and 700 μL ddH20 in triplicate. Background absorbance was measured at a wavelength of 412 nm once every 30 s for 5 min. Next, 10 mM oxaloacetate (50 μL) was added, and each cuvette was again read at 412 nm once every 30 s for 5 min. Data points were fit in a linear fashion for both readings, and slopes were determined. The slope prior to the addition of oxaloacetate was subtracted from the slope that was measured after the addition of oxaloacetate.

### 2.8 Statistics

Data were checked for normality using the Anderson‐Darling test. Normally distributed data were analyzed using an unpaired two‐tailed *t*‐test to determine differences between the means of the two groups (early follicular vs. luteal) and non‐normally distributed data were tested using a two‐tailed Mann–Whitney *U* test. Correlations were tested using a two‐tailed Pearson product–moment correlation test. The figure legends of each figure indicate the test used (e.g., *t*‐test or Mann–Whitney *U* test) as well as the sample size for each test. Hedges' *g* statistics, with bias correction for sample size, were calculated to report effect sizes (Hedges & Olkin, 1985). Data reported in the Results section give the *p* value of the difference in means, difference in means (early follicular compared to luteal), 95% confidence interval of the difference of means, and Hedges' *g*. For correlations, data are given as Pearson's *r*, 95% confidence interval of Pearson's *r*, and the *p* value of the correlation. Presented data are shown in the bar graphs as means ± one standard deviation, with data points representing individual data points. The scatterplots show each data point, with the trend lines demonstrating lines of best fit. Significant differences were tested with an *α* level of 0.05, established a priori. The Anderson‐Darling normality tests, unpaired *t*‐tests, Mann–Whitney *U* tests, and Pearson's correlation tests were run using GraphPad Prism (version 10.4.1). The 95% confidence intervals and Hedges' *g* statistics were calculated using Microsoft Excel (version 16.97).

## 3 RESULTS

### 3.1 Participant characteristics

Participant characteristics are reported in Table 1. No differences between groups were observed in menstrual cycle length, participant age, height, body mass, or body mass index (*p* > 0.05).

### 3.2 Mitochondrial respiration

**FIGURE 1:**
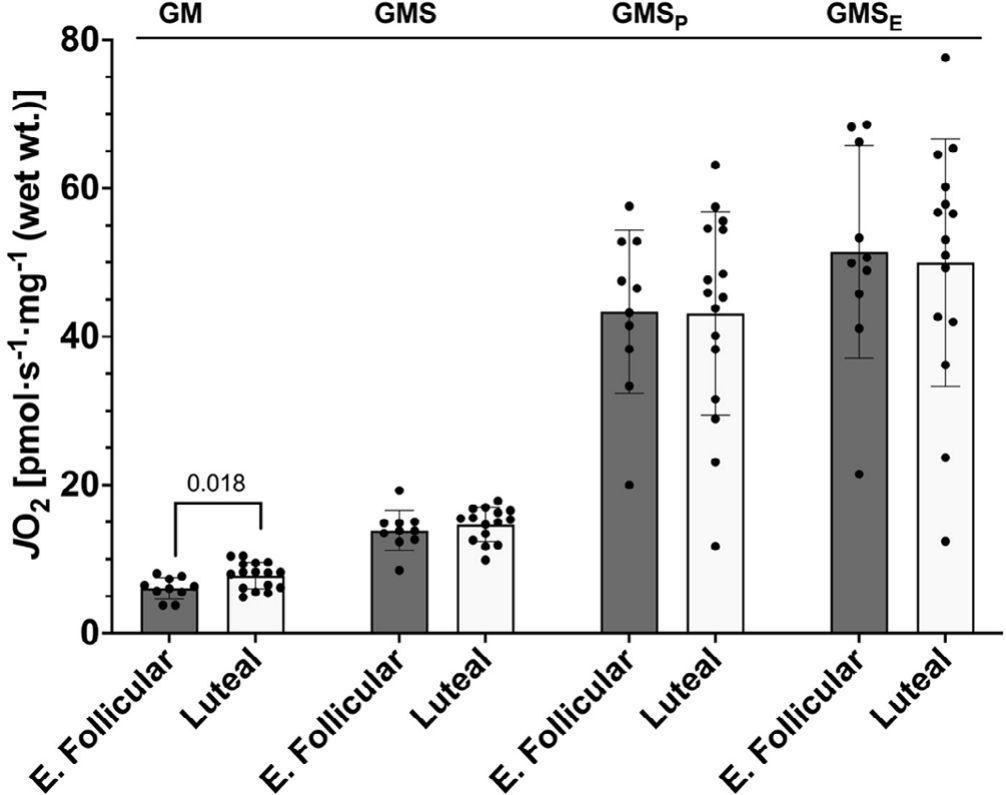
Mitochondrial respiration using NADH‐linked substrates and succinate. G, glutamate; M, malate; S, succinate; _P_, ADP; _E_, FCCP; E. Follicular, early follicular. Bars represent mean ± standard deviation, dots represent individual data points; early follicular *n* = 10, luteal *n* = 15 (GMS and GMS_E_), luteal *n* = 16 (GM and GMS_P_). Means were tested using unpaired *t*‐tests. Values above bars indicate *p* value when significantly different (*p* < 0.05).

To determine the influence of menstrual cycle phase on skeletal muscle mitochondrial function, muscle biopsies from the vastus lateralis muscle were taken from participants during either the early follicular phase or luteal phase of the menstrual cycle. The first protocol examined respiration when supported by NADH‐linked substrates and succinate. We began by testing the ability of mitochondria to consume O_2_ under non‐phosphorylating conditions without ADP present (i.e., LEAK respiration) supported by glutamate and malate (GM) and after the addition of succinate (GMS). GM LEAK respiration was significantly higher in muscle sampled during the luteal phase (*p* = 0.018, difference in means = −1.72 pmol O_2_·s^−1^·mg^−1^, 95% CI [−0.31, −3.12], Hedges' *g* = −0.99) (Figure 1). With succinate (GMS LEAK), O_2_ consumption in the early follicular group was no different from the luteal phase (*p* = 0.436, difference in means = −0.79 pmol O_2_·s^−1^·mg^−1^, 95% CI [−2.86, 1.28], Hedges' *g* = −0.31) (Figure 1). Maximal coupled respiration was then tested with the titration of ADP to the substrate solution of glutamate, malate, and succinate (GMS_P_). Maximal coupled mitochondrial respiration in the muscle from the luteal phase was not significantly different from the early follicular phase (*p* = 0.965, difference in means = 0.22 pmol O_2_·s^−1^·mg^−1^, 95% CI [−10.41, 10.85], Hedges' *g* = 0.02) (Figure 1). No significant differences between menstrual cycle phases were observed during uncoupled maximal respiration using glutamate, malate, succinate, ADP, with FCCP (GMS_E_) (*p* = 0.819, difference in means = 1.49 pmol O_2_·s^−1^·mg^−1^, 95% CI [−11.84, 14.82], Hedges' *g* = 0.09) (Figure 1). To assess mitochondrial outer membrane integrity, we also evaluated the cytochrome‐*c* response during Protocol 1. The mean increase in *J*O_2_ for the early follicular group was 4.42 ± 3.7% and for the luteal group it was 2.17 ± 5.1%, with no significant differences between them (*p* = 0.1197, difference in means = −2.25%, 95% CI [−0.76, 5.27], Hedges' *g* = 0.94).

**FIGURE 2:**
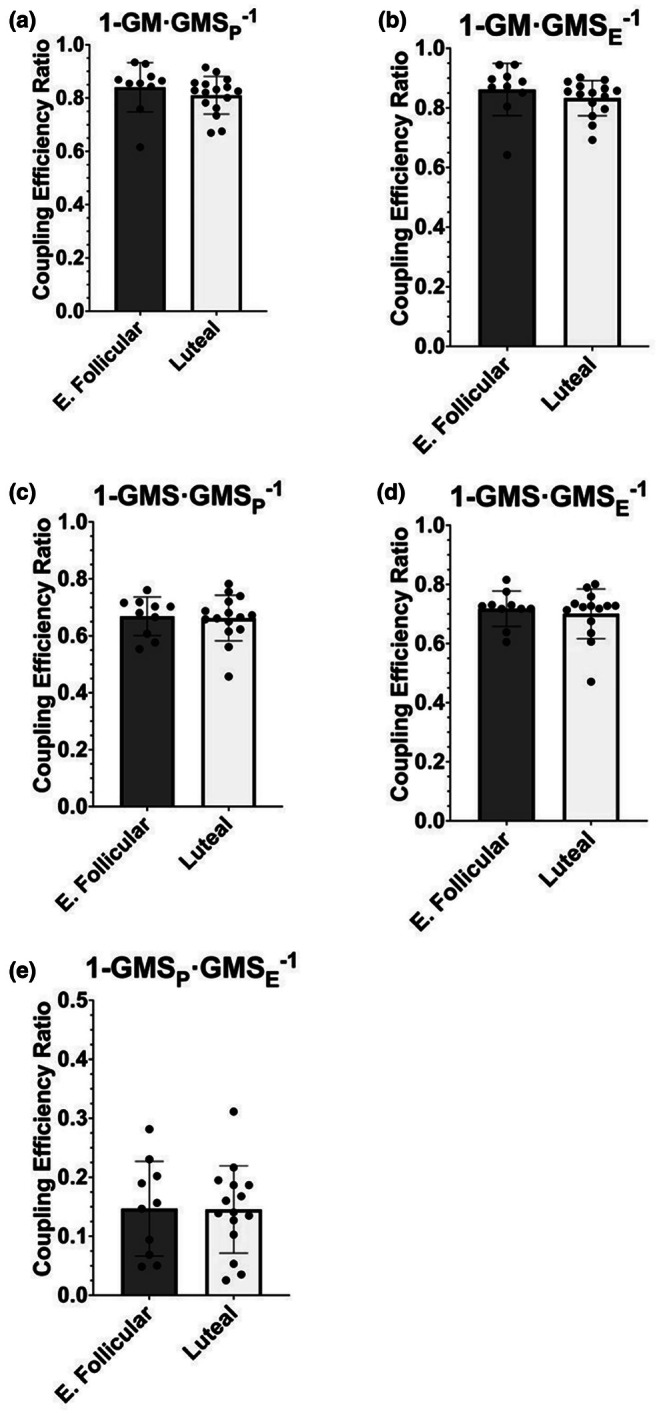
Mitochondrial coupling efficiency ratios for respiration using NADH‐linked substrates and succinate. G, glutamate; M, malate; S, succinate; _P_, ADP; _E_, FCCP; E. Follicular, early follicular. Bars represent mean ± standard deviation; dots represent individual data points: early follicular *n* = 10; luteal *n* = 14 (1‐GMS·GMS_E_^−1^), luteal *n* = 15 (1‐GM·GMS_E_^−1^, 1‐GMS·GMS_P_^−1^, and 1‐GMS_P_·GMS_E_^−1^), and luteal *n* = 16 (1‐GM·GMS_P_^−1^). Means were tested using unpaired *t*‐tests except for 1‐GM·GMS_E_^−1^, 1‐GM·GMS_P_^−1^, and 1‐GMS·GMS_E_^−1^, which were tested with Mann–Whitney *U* tests.

We then evaluated the coupling efficiency of mitochondrial function between the follicular and luteal phases using coupling efficiency ratios for respiration supported by NADH‐linked substrates and succinate. We observed no differences between groups with 1‐GM LEAK·GMS_P_^−1^ (*p* = 0.121, difference in means = −0.032, 95% CI [−0.14, 0.09], Hedges' *g* = 0.33) (Figure 2a) or 1‐GM LEAK·GMS_E_^−1^ (*p* = 0.091, difference in means = 0.029 JO_2_, 95% CI [−0.0006, 0.088], Hedges' *g* = 0.39) (Figure 2b). For GMS LEAK and maximal coupled respiration (GMS_P_) (1‐GMS LEAK·GMS_P_^−1^) there were no differences (*p* = 0.833, difference in means = 0.006, 95% CI [−0.057, 0.071], Hedges' *g* = 0.08) (Figure 2c) and similarly, the ratios of 1‐GMS LEAK·GMS_E_^−1^ were not different between the luteal phase and early follicular phase (*p* = 0.931, difference in means = 0.002, 95% CI [−0.037, 0.057], Hedges' *g* = 0.22) (Figure 2d). Finally, there were no differences between the groups in coupling efficiency for maximal coupled respiration (GMS_P_) and maximal uncoupled respiration measures (GMS_E_) (1‐GMS_P_·GMS_E_^−1^) (*p* = 0.965, difference in means = 0.0014, 95% CI [−0.064, 0.067], Hedges' *g* = 0.02) (Figure 2e).

**FIGURE 3:**
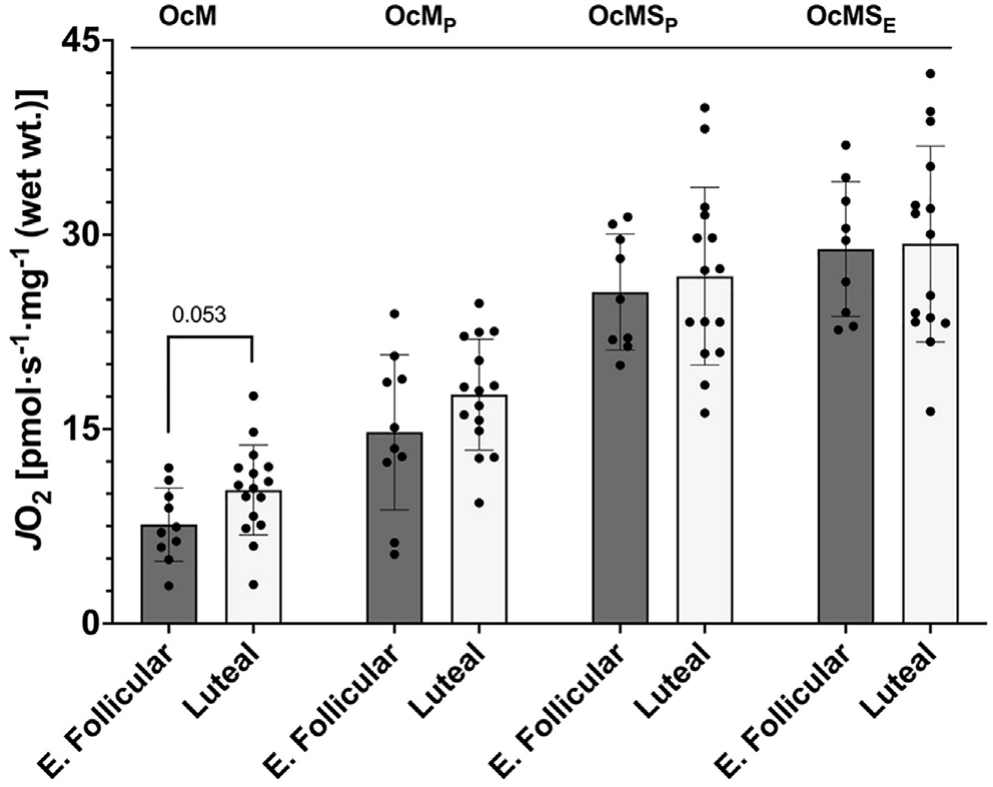
Mitochondrial respiration using fatty acid oxidation with NADH‐linked substrates and succinate. Oc, octanoylcarnitine; M, malate; S, succinate; _P_, ADP; _E_, FCCP; E. Follicular, early follicular. Bars represent mean ± standard deviation; dots represent individual data points: early follicular *n* = 9 (OcMS_P_ and OcMS_E_), early follicular *n* = 10 (OcM and OcM_P_), luteal *n* = 15(OcM_P_, OcMS_P_, and OcMS_E_), and luteal *n* = 16(OcM). Means were tested using unpaired *t*‐tests. Values above bars indicate *p* value.

Our second respiration protocol evaluated the electron transfer system when supported by fatty acid oxidation with NADH‐linked substrates and succinate. First, we assessed LEAK respiration (octanoylcarnitine and malate, OcM LEAK) but detected no significant differences between the phases (*p* = 0.053, difference in means = −2.66 pmol O_2_·s^−1^·mg^−1^, 95% CI [−5.37, 0.04], Hedges' *g* = −0.80) (Figure 3). We then evaluated coupled respiration with the titration of ADP (OcM_P_). Mitochondria from the luteal phase consumed the same rate of O_2_ as the early follicular phase in this condition (*p* = 0.167, difference in means = −2.92 pmol O_2_·s^−1^·mg^−1^, 95% CI [−7.16, 1.32], Hedges' *g* = −0.56) (Figure 3). When octanoylcarnitine, malate, ADP, and succinate (OcMS_P_) were used to support maximal coupled respiration, there was no significant difference in respiration (*p* = 0.64, difference in means = −1.21 pmol O_2_·s^−1^·mg^−1^, 95% CI [−6.54, 4.12], Hedges' *g* = −0.19) (Figure 3). Uncoupled maximal respiration was determined with the titration of FCCP to octanoylcarnitine, malate, ADP, and succinate (OcMS_E_). Uncoupled maximal mitochondrial respiration was the same between the luteal phase and early follicular phases (*p* = 0.889, difference in means = −0.4 pmol O_2_·s^−1^·mg^−1^, 95% CI [−6.34, 5.54], Hedges' *g* = −0.06) (Figure 3). The cytochrome‐*c* response was also assessed after Protocol 2. The early follicular mean increase was 4.46 ± 6.2%, and the mean increase for the luteal group was 2.61 ± 4.8%; there were no differences between groups (*p* = 0.2284, difference in means = 1.94%, 95% CI [−1.35, 5.05], Hedges' *g* = 0.45).

**FIGURE 4:**
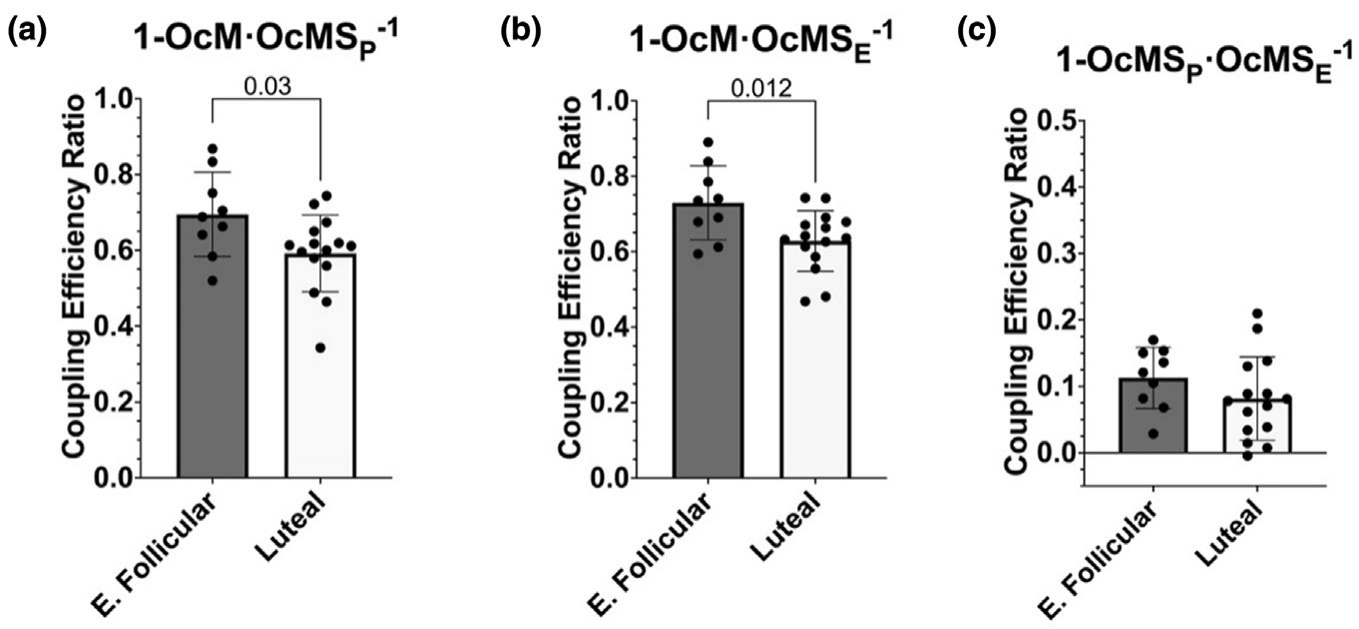
Mitochondrial coupling efficiency ratios for respiration using fatty acid oxidation, NADH‐linked substrates, and succinate. Oc, octanoylcarnitine; M, malate; S, succinate; _P_, ADP; _E_, FCCP; E. Follicular, early follicular. Bars represent mean ± standard deviation; dots represent individual data points; early follicular *n* = 9, luteal *n* = 15. Means were tested using unpaired *t*‐tests. Values above bars indicate *p* value when significantly different (*p* < 0.05).

Lastly, we evaluated three coupling efficiency ratios using respiration driven by fatty acid oxidation, malate, and succinate. The 1‐OcM LEAK over max coupled (1‐OcM·OcMS_P_^−1^) ratio was 14% higher in the early follicular phase compared to the luteal phase (*p* = 0.03 difference in means = 0.103, 95% CI [0.011, 0.194], Hedges' *g* = 0.95) (Figure 4a). Likewise, the ratio of 1‐OcM LEAK over max uncoupled (OcMS_E_) (1‐OcM·OcMS_E_^−1^) was 13% higher in the early follicular mitochondria than the luteal (*p* = 0.012, difference in means = 0.101, 95% CI [0.025, 0.177], Hedges' *g* = 1.12) (Figure 4b). However, the maximal coupled (OcMS_P_) over max uncoupled (OcMS_E_) (1‐OcMS_P_·OcMS_E_^−1^) ratio was not different between phases (*p =* 0.211, difference in means = 0.031, 95% CI [−0.019, 0.081], Hedges' *g* = 0.53) (Figure 4c).

### 3.3 Mitochondrial H_2_O_2_ production

**FIGURE 5:**
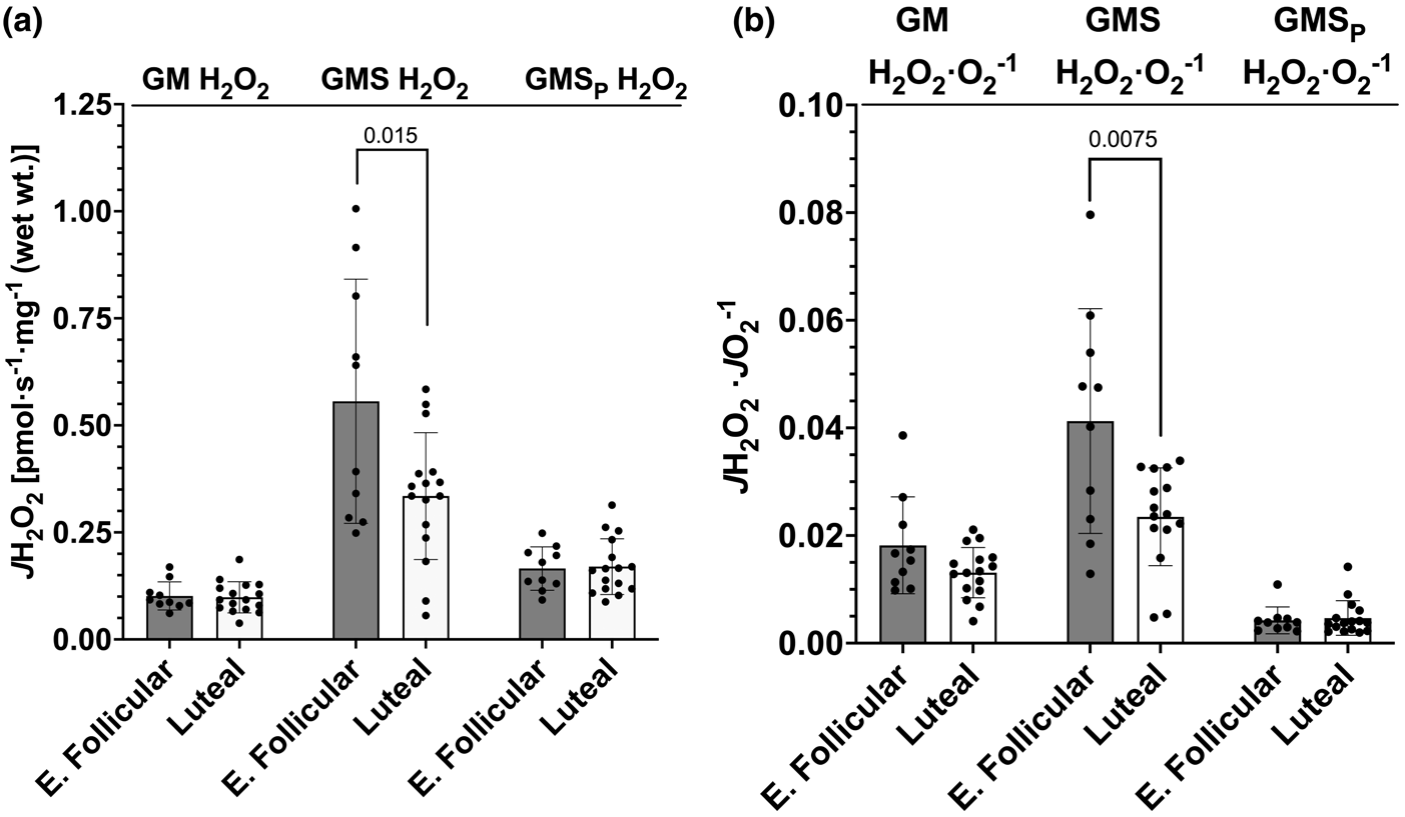
Mitochondrial H_2_O_2_ production. (a) absolute *J*H_2_O_2_. (b) *J*H_2_O_2_·*J*O_2_^−1^. G, glutamate; M, malate; S, succinate; _P_, ADP; E. Follicular, early follicular. Bars represent mean ± standard deviation; dots represent individual data points; early follicular *n* = 10, luteal *n* = 16 for all comparisons except GMS *J*H_2_O_2_·*J*O_2_^−1^ luteal *n* = 15. Means for absolute H_2_O_2_, GM *J*H_2_O_2_·*J*O_2_‐1, and GMS_P_ *J*H_2_O_2_·*J*O_2_‐1 were tested using unpaired *t*‐tests; means for GMS *J*H_2_O_2_·*J*O_2_^−1^ were tested with a Mann–Whitney *U* test. Values above bars indicate *p* value when significantly different (*p* < 0.05).

To investigate if menstrual cycle phase influenced mitochondrial reactive oxygen species production, we determined absolute H_2_O_2_ emission in skeletal muscle samples. Glutamate and malate (GM) driven H_2_O_2_ production was similar for both phases of the menstrual cycle (*p* = 0.826, difference in means = 0.003 pmol H_2_O_2_·s^−1^·mg^−1^, 95% CI [−0.026, 0.032], Hedges' *g* = 0.09) (Figure 5). However, when succinate was used along with glutamate and malate (GMS) as a substrate to drive H_2_O_2_ production, the early follicular phase produced significantly more H_2_O_2_ than the luteal phase (*p* = 0.015, difference in means = 0.22 pmol H_2_O_2_·s^−1^·mg^−1^, 95% CI [0.009, 0.433], Hedges' *g* = 1.02) (Figure 5). When submaximal concentrations of ADP (12.5 μM) were then added to glutamate, malate, and succinate (GMS_P_) to assess H_2_O_2_ production, we observed no difference between phases (*p* = 0.852, difference in means = −0.005 pmol H_2_O_2_·s^−1^·mg^−1^, 95% CI [−0.055, 0.045], Hedges' *g* = −0.07) (Figure 5). Similar findings were observed when H_2_O_2_ emission was normalized to O_2_ consumption for each respiration state. We observed a significantly higher GMS *J*H_2_O_2_·*J*O_2_^−1^ in the early follicular phase compared to the luteal phase (*p* = 0.0075, difference in means = 0.0178 *J*H_2_O_2_·*J*O_2_^−1^, 95% CI [0.0052, 0.0303], Hedges' *g* = 1.32) (Figure 5). There were no differences between either phase for GM *J*H_2_O_2_·*J*O_2_^−1^ (*p* = 0.0708, difference in means = −0.005 *J*H_2_O_2_·*J*O_2_^−1^, 95% CI [−0.0005, 0.0106], Hedges' *g* = 0.89) or GMS_P_ *J*H_2_O_2_·*J*O_2_^−1^ (*p* = 0.8564, difference in means = −0.00043 *J*H_2_O_2_·*J*O_2_^−1^, 95% CI [−0.003, 0.002], Hedges' *g* = −0.27) (Figure 5).

### 3.4 Citrate synthase activity

**FIGURE 6:**
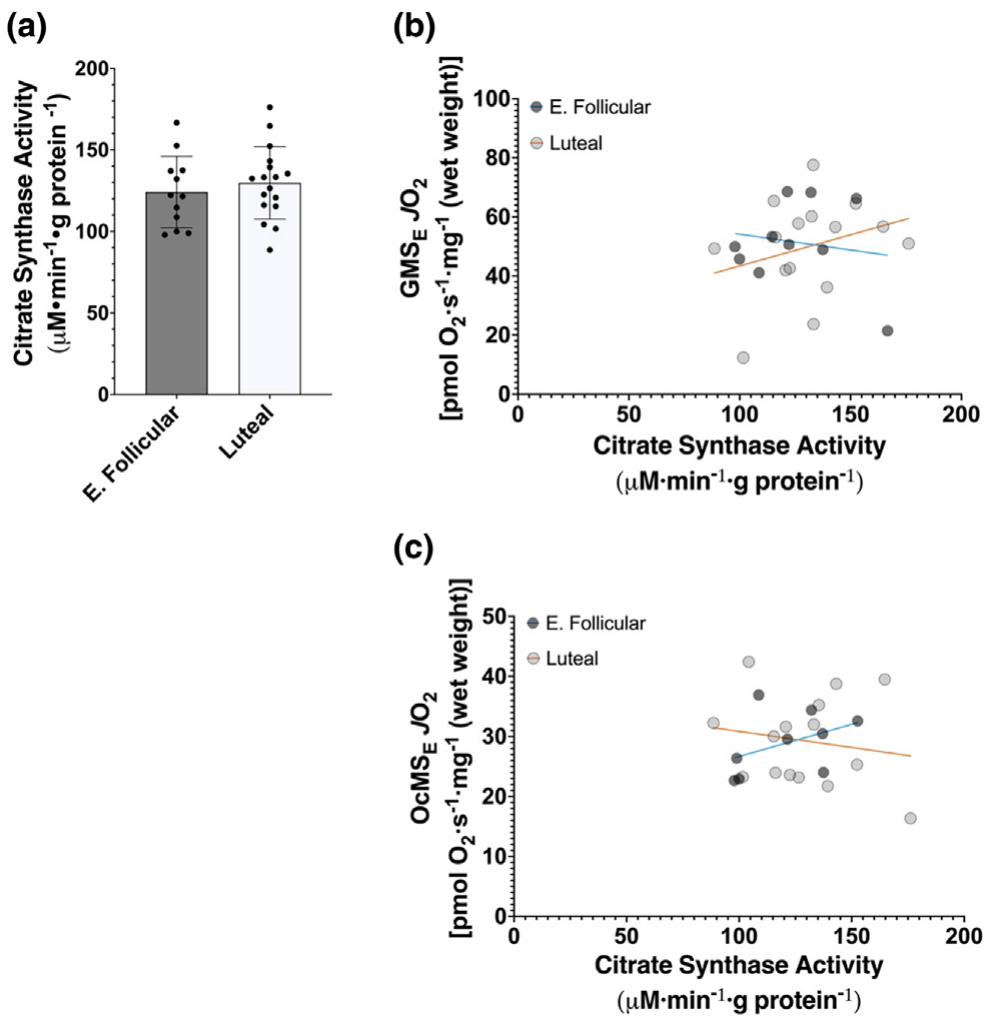
(a) Citrate synthase activity. E. Follicular, early follicular. Bars represent mean ± standard deviation, dots represent individual data points; early follicular *n* = 12, luteal *n* = 17. Means were tested using an unpaired *t*‐test. (b) Correlation between maximal uncoupled respiration supported by NADH‐linked substrates and succinate (GMS_E_) and citrate synthase activity. E. Follicular: r = −0.17, 95% CI [−0.72, 0.52], *p* = 0.6547, *n* = 10 pairs; Luteal: r = 0.29, 95% CI [−0.26, 0.7], *p* = 0.2998, *n* = 15 pairs. (c) Correlation between maximal uncoupled respiration supported by fatty acid oxidation with NADH‐linked substrates and succinate (OcMS_E_) and citrate synthase activity. E. Follicular: *r* = 0.42, 95% CI [−0.34, 0.85], *p* = 0.2643, *n* = 9 pairs; Luteal: *r* = −0.17, 95% CI [−0.63, 0.38], *p* = 0.5497, *n* = 15 pairs. Trend lines represent best fits; correlations were tested using the Pearson product–moment correlation test.

We observed no difference in citrate synthase activity between menstrual cycle phases (*p* = 0.507, difference in means = −5.7 μM·min^−1^·gram protein^−1^, 95% CI [−22.8, 11.4], Hedges' *g* = −0.25) (Figure 6a). We also examined if citrate synthase activity was correlated with maximal uncoupled respiration for both respiration protocols. In muscle sampled during the early follicular phase, maximal uncoupled respiration supported by NADH‐linked substrates and succinate was not correlated with citrate synthase activity (*r* = −0.17, 95% CI [−0.72, 0.52], *p* = 0.6547) (Figure 6b). Likewise, no significant correlation was found in mitochondria from the luteal group (*r* = 0.29, 95% CI [−0.26, 0.7], *p* = 0.2998) (Figure 6b). We also assessed this association with maximal uncoupled respiration supported by fatty acid oxidation with NADH‐linked substrates and succinate and found similar results, no significant correlations in either phase of the menstrual cycle (early follicular: *r* = 0.42, 95% CI [−0.34, 0.85], *p* = 0.2643 and luteal: *r* = −0.17, 95% CI [−0.63, 0.38], *p* = 0.5497) (Figure 6c).

## 4 DISCUSSION

Previous research has demonstrated that mitochondrial function is influenced by female sex hormones (Klinge, 2020; Ventura‐Clapier et al., 2019; Yoh et al., 2023). However, prior studies have primarily used rodent models, ovariectomy (OVX), and/or exogenous sex hormone formulations and dosages that may not accurately represent typical human physiology. Given that sex hormones naturally fluctuate throughout the menstrual cycle, we sought to determine if patterns of influence were observable in the skeletal muscle mitochondria of young, healthy females during two phases of the menstrual cycle. We evaluated the menstrual cycle influence on mitochondria during the early follicular phase due to its relatively low circulating levels of estradiol and progesterone. The luteal phase was chosen for its elevated levels of both estradiol and progesterone. We evaluated mitochondrial function using two different methods, one to evaluate general maximum respiratory capacity and one to measure reactive oxygen species emission. Overall, we observed a subtle influence on LEAK respiration but no effect on citrate synthase activity or maximal respiration supported by glutamate, malate, and succinate (GMS) or fatty acid oxidation with malate and succinate (OcMS). We do report differences in mitochondrial coupling efficiency of fatty acid driven respiration, indicative of differences in efficiency. Lastly, we also observed an influence of the menstrual cycle on H_2_O_2_ emission when supported by GMS.

### 4.1 Mitochondrial respiration

When mitochondrial function was assessed during the luteal phase, GM LEAK respiration was significantly elevated when supported with glutamate and malate. These substrates provide reducing equivalents for Complex I of the electron transport chain. When the substrate succinate is then added to the GM LEAK state, Complex I and II are both utilized in a condition referred to as GMS LEAK. In this condition, however, we observed no differences in respiration between menstrual cycle phases. Thus, the differences between the phases seemed to be more dependent upon Complex I and not Complex II supported respiration. Our results indirectly support the previous observations of others that elevated estradiol improved respiration through Complex I and had little effect on Complex II (Torres, Kew, et al., 2018). Using ovariectomized mice, administration of exogenous estradiol improved both the respiration driven by Complex I substrates (glutamate/malate LEAK and glutamate/malate/ADP coupled) and additionally, estradiol administration increased the relative specific enzyme activity of Complex I but had no impact on the relative specific enzyme activity of Complex II. A recent report assessing muscle mitochondrial function between postmenopausal women that received hormone replacement therapy (estradiol and progestin) with those that did not does not align with our findings. They found no difference in maximal Complex I supported respiration (malate/pyruvate/glutamate/ADP) between the two groups. They did, however, observe significant increases in maximal Complex I + II supported respiration (malate/pyruvate/glutamate/ADP/succinate) as well as maximal uncoupled respiration (FCCP) (Kleis‐Olsen et al., 2024). One potential explanation for the discrepancy in findings of this study and our data is participant age. The participants in their study were postmenopausal women with mean ages for control participants of 56 ± 4 years and treatment participants of 55 ± 5 years. Our participants' mean ages were 20 ± 2 years for the early follicular phase participants and 22 ± 2 years for the luteal phase participants. This disparity in age makes it difficult to tease out the potential influences of aging on mitochondrial respiration from the altered hormone profiles of postmenopausal women with and without hormone replacement therapy.

Numerous studies have found that during exercise, women rely on fatty acid oxidation in skeletal muscle to a greater extent than men do, which has been linked to the effects of estrogens (Fu et al., 2009; Knechtle et al., 2004; Riddell et al., 2003; Tarnopolsky, 2000). In studies where males received estradiol therapy, a shift in macronutrient reliance was observed towards greater dependence on lipid metabolism (Fu et al., 2009; Hamadeh et al., 2005). These same results have been observed in primary myotubes from male participants (Salehzadeh et al., 2011). Estradiol depletion has also been linked to impaired lipid metabolism and accumulation of adipose tissue (D'Eon et al., 2005; Kamei et al., 2005). Recently, many have investigated peroxisome proliferator‐activated receptors (PPAR) and their role in regulating genes involved in fatty acid oxidation. PPARα and PPARδ are both upregulated in the presence of estradiol (Campbell et al., 2003; Fu et al., 2009), which in turn increases gene expression of proteins involved in beta‐oxidation (Braun & Horton, 2001; D'Eon et al., 2005; Fu et al., 2009). In our study, we did not see significant differences between the fatty acid oxidation capacity of participants in the early follicular and luteal phases. We suspect three potential reasons we did not see differences in fatty acid oxidation in participants at these two points in the menstrual cycle. First, in studies where direct effects of estradiol alone are studied, changes in fatty acid oxidation are much more likely to be observed than in the model we evaluated, which included a complex interaction of estrogens and progesterone among other hormones. Campbell et al. found that treatment with both estradiol and progesterone produced less PPARα mRNA than when only estradiol was used (Campbell et al., 2003), suggesting that these two major hormones interact together to impact fatty acid oxidation capacity (Braun & Horton, 2001). Second, hormone levels in our participants were not altered nor controlled by drastic interventions such as those done in ovariectomized mouse studies. Our study observed the effect that the natural menstrual cycle has on various aspects of mitochondrial bioenergetics in healthy females. Third, Torres et al. suggested that many of the proteins involved in fatty acid oxidation that are regulated by PPAR seem to have long‐lasting effects (Torres, Kew, et al., 2018). In their study, they saw that estradiol treatment in ovariectomized mice rescued insulin sensitivity, mitochondrial function, H_2_O_2_ emissions, and antioxidant activity to normal levels. However, fatty acid oxidation capacity in skeletal muscle was not restored with estradiol therapy. The finding that fatty acid oxidation remained low following treatment with estradiol suggests that there may be long‐term estradiol regulatory functions that are not restored over short durations of time (Campbell et al., 2003; Torres, Kew, et al., 2018). Therefore, there may not be sufficient time within the early follicular phase of the menstrual cycle to observe a significant decrease in fatty acid oxidation capacity before estradiol levels naturally rise again. Although we did not see differences in the fatty acid oxidation capacity of our two groups, our observations have application for studies on fatty acid metabolic function due to our use of a physiological model of the menstrual cycle without intervention.

Several studies have investigated sex differences in mitochondrial respiration (Cardinale et al., 2018; Ferguson et al., 2025; Miotto et al., 2018; Monaco et al., 2020; Montero et al., 2018). Like them, we failed to see any differences in maximal rates of oxygen consumption. These previous studies demonstrated that even if sex differences in mitochondrial respiration existed in submaximal conditions, once maximal respiration is induced experimentally, these differences disappeared. This growing body of literature indicates that maximal mitochondrial respiration may not be as sensitive to biological sex as submaximal respiration may be. One possible explanation is that maximal respiration can be viewed as a function of mitochondrial content and/or mitochondrial protein abundance, neither of which is believed to be particularly prone to acute changes. Our work supports this notion. We observed no differences between groups in citrate synthase activity, a biomarker of mitochondrial content in skeletal muscle (Larsen et al., 2012). We additionally examined if citrate synthase activity correlated with maximal respiration and saw no such relationship. Given that we reported no differences in maximal respiration, the lack of correlation is not surprising. Regardless, our findings regarding maximal respiration align with these previous reports. Importantly, though, unlike the previous investigations, this study only measured mitochondrial respiration of female participants in two distinct phases of the menstrual cycle with no other physiological interventions. Based on these previous publications, our findings regarding maximal respiration are not unexpected while also still being somewhat novel given the sex of our participants and the conditions we studied. Future work could further explore the resistance of maximal respiration to variables such as sex and hormone profiles.

### 4.2 Mitochondrial coupling efficiency

It should be noted that we did observe differences in two of the three coupling efficiency ratios we evaluated using fatty acid supported respiration data. Elevated coupling efficiency ratios using the 1‐Ratio convention indicate improved efficiency, or in other words, the absolute LEAK respiration values represent less of the overall capacity of the maximal coupled or uncoupled respiration states. In our data, respiration in mitochondria sampled during the early follicular phase was more efficiently coupled for both 1‐OcM LEAK·OcMS_P_^−1^ (max coupled) and 1‐OcM LEAK·OcMS_E_^−1^ (max uncoupled). Interestingly, as just discussed, there were no statistical differences in absolute fatty acid driven respiration between groups (Figure 2). However, OcM LEAK was 35% higher in the mitochondria from the luteal phase group, but this difference was not considered significant (*p* = 0.053) despite a “large” Hedges' *g* effect size (−0.8). As noted, this difference did not achieve statistical significance, but when factored over the maximal coupled (OcMS_P_) and maximal uncoupled (OcMS_E_) values, it impacts the efficiency coupling ratios. Taken in context of our other fatty acid respiration observations, we cannot say if these differences in coupling efficiency are meaningful. Future work could be done to better understand the influence of the menstrual cycle on the details of fatty acid supported respiration and coupling efficiency.

### 4.3 Production of reactive oxygen species

Notably, estradiol has been shown to regulate the redox balance in multiple cell types, including skeletal myofibers, by increasing expression and activity of antioxidant enzymes and preventing oxidative damage (Baltgalvis et al., 2010; Irwin et al., 2008; Munoz‐Castaneda et al., 2005; Nilsen et al., 2007; Nilsen & Brinton, 2004; Strehlow et al., 2003). Additionally, previous work has demonstrated that ovariectomized mice produce higher amounts of mitochondrial reactive oxygen species in muscle and that treatment with estradiol prevents this (Torres, Kew, et al., 2018). Others have demonstrated that mice with an estrogen receptor α knockout phenotype in muscle see elevated production of both H_2_O_2_ and O_2_^−^; they also report reductions in glutathione peroxidase 3 protein and mRNA as well as increases in protein carbonylation compared to control mice (Ribas et al., 2016). Torres et al. showed that estradiol localizes to the inner membrane, changing the microviscosity of the inner membrane (Torres, Kew, et al., 2018). They noted that in doing so, estradiol could potentially limit lipid peroxidation and preserve the function of the electron transport system. This observation was based on experiments performed in brain mitochondria by Irwin et al., who observed that administration of estradiol and progesterone reduced lipid peroxidation and electron leak (Irwin et al., 2008). Interestingly, these findings in rodents differ from observations in postmenopausal women that saw no influence of hormone replacement therapy on muscle mitochondrial H_2_O_2_ production (Kleis‐Olsen et al., 2024). The authors speculated that because the hormone replacement therapy used in their experiments contained both estradiol and progestin, there could have been a countering effect by each hormone. Indeed, previous work has demonstrated that progesterone can increase H_2_O_2_ emission from mitochondria (Kane et al., 2011) while, as noted above, estradiol can decrease reactive oxygen species generation from mitochondria (Torres, Kew, et al., 2018). It is also worth noting that the aged condition of post‐menopausal women could be a factor in the differential findings related to redox biology.

The influence of estradiol and progesterone on membrane viscosity may also affect other aspects of mitochondrial respiration, like LEAK respiration. This should not be surprising given that the experimental conditions used to induce LEAK respiration also favor reactive oxygen species (ROS) production, specifically the presence of an elevated proton motive force in the absence of ATP synthesis. We observed that the luteal phase had higher LEAK respiration for GM and OcM, as the difference observed for OcM LEAK approached significance (*p* = 0.053) and also had a large effect size (Hedges' *g* = −0.8). As discussed previously, recent reports demonstrate that submaximal respiration parameters, like LEAK, appear to be more susceptible to variables like biological sex than maximal respiration. These differences in LEAK respiration could also potentially be attributed to hormonally driven changes in membrane viscosity where protons are more easily allowed to move back across the membrane. Additionally, changes in membrane viscosity could also affect the transport of other substrates like ADP. However, our experiments were not set up to test this specifically.

In our measurements, only H_2_O_2_ production with GMS revealed any significant difference between menstrual cycle phases. The other two measurements we made of H_2_O_2_ emission were similar between phases of the menstrual cycle. The observation that GMS generated the most ROS is somewhat expected. In this condition (GMS), the proton motive force is believed to be at its highest, and ROS production has been shown to be directly influenced by an elevated proton motive force (Lambert & Brand, 2004). As mentioned previously, estradiol can localize to the inner membrane and influence ROS generation (Torres, Kew, et al., 2018). Considering that the luteal phase was associated with less emission of H_2_O_2_, our findings support this observation along with other work done in rodents that have demonstrated an influence of estradiol on ROS production (Irwin et al., 2008; Ribas et al., 2016; Torres, Kew, et al., 2018).

### 4.4 Respiration buffer composition

Creatine and its metabolic counterpart, phosphocreatine, are essential bioenergetic components found in skeletal muscle. They are often used experimentally in mitochondrial respiration protocols to examine mitochondrial function with more physiological concentrations of ATP/ADP ratios (Fisher‐Wellman et al., 2018; McFaline‐Figueroa et al., 2023). The inclusion of creatine in respiration buffers is beneficial when evaluating mitochondrial function when lower concentrations of ADP are critical and has even been shown to enhance oxygen consumption (Kay et al., 2000; Walsh et al., 2001). However, once ADP concentrations become saturating, the influence of creatine on mitochondrial respiration ceases (Walsh et al., 2001). Our protocol was designed to examine maximal respiration as well as H_2_O_2_ emission between two phases of the menstrual cycle. As such, our protocol dictated the use of saturating concentrations of ADP to experimentally induce the conditions necessary to obtain these data. Given this, the presence of creatine in our respiration buffer would have been inconsequential and thus omitted from our respiration buffer. Certainly, future work could explore the influence of the menstrual cycle on additional mitochondrial respiration variables, particularly those sensitive to the presence of creatine. In fact, a recent report investigated the use of a creatine kinase clamp method to examine mitochondrial respiration in skeletal muscle with a more physiological ratio of ATP/ADP (McFaline‐Figueroa et al., 2023). This method, developed and optimized by several previous groups, relies on a more physiological ratio of ATP/ADP which is a known regulator of critical mitochondrial functions (Fisher‐Wellman et al., 2018; Glancy et al., 2013; McLaughlin et al., 2020; Messer et al., 2004; Schmidt et al., 2021). From an applied perspective, future work could also investigate the role of creatine on mitochondrial function throughout the menstrual cycle given the cyclical fluctuations of sex hormones and creatine kinase activity (Smith‐Ryan et al., 2021).

### 4.5 Limitations

Our cross‐sectional study design warrants a brief mention. When compared to a repeated measures design, the current study admittedly has lower statistical power. However, the current design benefits from a proper randomization protocol that ensured participant interchangeability while also being able to aptly describe potential variability between participants without concern of an order effect or insufficient washout periods. Given our research question, capturing natural inter‐person variability seems appropriate and facilitates generalizing the findings to a wider population. We also acknowledge the lack of plasma estradiol and progesterone concentrations, both of which are known to be elevated during the luteal phase and at their lowest relative concentrations during the early follicular phase. While these data could have been informative, we are confident in the phase and timing of the menstrual cycles given our procedures to track the menstrual cycle phase. Importantly, our research question is not specifically focused on the specific actions of estradiol or progesterone per se, but rather mitochondrial energetics across two menstrual cycle phases. Lastly, a recent report attributes sex differences in mitochondrial respiration to differences in aerobic capacity (Ferguson et al., 2025). We did not measure aerobic fitness in participants. However, we did exclude participants that exercised more than 120 min a week, which would at least minimize differences in aerobic capacity arising from regular sustained physical activity. We also measured citrate synthase activity, which serves as a proxy for mitochondrial content, and we saw no differences between groups.

## 5 CONCLUSION

In conclusion, our findings demonstrate that the menstrual cycle phase does not have a significant overall influence on GMS supported respiration or OcM supported respiration. There also does not appear to be any influence on citrate synthase activity between menstrual phases. However, differences were detected in GM LEAK, fatty acid supported coupling efficiency ratios, and H_2_O_2_ emission. Future research could attempt to better understand their potential implications. Overall, these data suggest that menstrual cycle phase may be an essential variable to control for in future studies that assess certain aspects of mitochondrial function.

## FUNDING INFORMATION

Funding was provided by an internal award to W. Bradley Nelson from the College of Life Sciences, Brigham Young University, supported by a gift from Ira and Mary Lou Fulton.

## CONFLICT OF INTEREST STATEMENT

The authors have no conflicts of interest to disclose.

## Data Availability

The data that support the findings of this study are available from the corresponding author upon reasonable request.
